# Validating the use of Hospital Episode Statistics data and comparison of costing methodologies for economic evaluation: an end-of-life case study from the Cluster randomised triAl of PSA testing for Prostate cancer (CAP)

**DOI:** 10.1136/bmjopen-2016-011063

**Published:** 2016-04-29

**Authors:** Joanna C Thorn, Emma L Turner, Luke Hounsome, Eleanor Walsh, Liz Down, Julia Verne, Jenny L Donovan, David E Neal, Freddie C Hamdy, Richard M Martin, Sian M Noble

**Affiliations:** 1 School of Social and Community Medicine, University of Bristol, Bristol, UK; 2 Public Health England National Cancer Intelligence Network, Bristol, UK; 3 Nuffield Department of Surgical Sciences, John Radcliffe Hospital, Oxford, UK

**Keywords:** resource use, cost-effectiveness analysis, economic evaluation, costing methodology, hospital episode statistics, validation

## Abstract

**Objectives:**

To evaluate the accuracy of routine data for costing inpatient resource use in a large clinical trial and to investigate costing methodologies.

**Design:**

Final-year inpatient cost profiles were derived using (1) data extracted from medical records mapped to the National Health Service (NHS) reference costs via service codes and (2) Hospital Episode Statistics (HES) data using NHS reference costs. Trust finance departments were consulted to obtain costs for comparison purposes.

**Setting:**

7 UK secondary care centres.

**Population:**

A subsample of 292 men identified as having died at least a year after being diagnosed with prostate cancer in Cluster randomised triAl of PSA testing for Prostate cancer (CAP), a long-running trial to evaluate the effectiveness and cost-effectiveness of prostate-specific antigen (PSA) testing.

**Results:**

Both inpatient cost profiles showed a rise in costs in the months leading up to death, and were broadly similar. The difference in mean inpatient costs was £899, with HES data yielding ∼8% lower costs than medical record data (differences compatible with chance, p=0.3). Events were missing from both data sets. 11 men (3.8%) had events identified in HES that were all missing from medical record review, while 7 men (2.4%) had events identified in medical record review that were all missing from HES. The response from finance departments to requests for cost data was poor: only 3 of 7 departments returned adequate data sets within 6 months.

**Conclusions:**

Using HES routine data coupled with NHS reference costs resulted in mean annual inpatient costs that were very similar to those derived via medical record review; therefore, routinely available data can be used as the primary method of costing resource use in large clinical trials. Neither HES nor medical record review represent gold standards of data collection. Requesting cost data from finance departments is impractical for large clinical trials.

**Trial registration number:**

ISRCTN92187251; Pre-results.

**Strengths and limitations of this study**

- Resource-use data collection from medical records was both meticulous and comprehensive, allowing us to assess the suitability of the Hospital Episode Statistics (HES) inpatient data set for costing purposes in an economic evaluation alongside a clinical trial.
- The final year of life was accurately identified by analysing data retrospectively.
- The study focuses on a single clinical area only.
- Problems with using HES data include its limited geographical coverage, lack of sensitivity to details of procedures, uncertain definition of a day case and administrative changes in past years.

## Introduction

While guidelines have long recommended reporting resource use and unit costs separately,^1^ ^2^ there is little advice about appropriate costing methodology. There is consistency in the basic principles of costing, but guidelines contain disagreements about how best to apply costs to resource use.^3^ There has been substantial variation in costing methods applied within cost-effectiveness analyses (CEA) and results may be affected by the costing methods used.^4^ Attempts to improve the standardisation of costing methods to ensure comparability across studies^5–7^ have been hampered by a lack of transparency in reporting, with many studies reporting costing methodologies in an opaque fashion.^8^

There are necessary trade-offs between research resources available and the accuracy of the costs that can be obtained. Typically, health economists have collected information on resources consumed by extracting data from medical records or by asking patients themselves to supply data. Similarly, cost data relating to resource use identified within a trial is commonly obtained by asking finance departments in the National Health Service (NHS) trusts to supply data; over half the Health Technology Assessment (HTA) studies that reported an economic evaluation used costs from local sources.^9^ However, these can be time consuming and costly processes, and may not represent the best use of research resources. Increased access to routinely collected data in the UK, such as the NHS reference costs and the Hospital Episode Statistics (HES) data set in England,^10^ ^11^ is affording researchers opportunities for obtaining costs in healthcare more readily. For example, HES data can be used to identify relevant resource use, while the NHS reference costs can potentially be used in place of data from finance departments. Each hospital inpatient stay recorded in HES is assigned a healthcare resource group (HRG) code, with HRGs representing collections of procedures that typically use similar levels of resources (based on the diagnosis-related groups (DRGs) used in the USA). However, data such as HES are primarily recorded for the purposes of administering the health service and, as such, are not specifically designed for research purposes; therefore, there may be limitations to the usefulness of the data. In addition, the data have not been validated for use in economic evaluation costing studies, and therefore it is not possible to say how accurate or complete they are; researchers and policymakers cannot be confident the results reported are correct without some form of validation having been undertaken.^12^

Healthcare at the end of life is a topic of current national interest, with the National Institute for Health and Care Excellence (NICE) having issued additional guidance for considering interventions that might increase the length of life for patients close to death.^13^ A sharp increase in the costs associated with healthcare resources consumed is typically observed in the last months before death,^14–16^ with men dying from prostate cancer incurring their highest costs close to diagnosis and death.^17^

In this study, the aim was to compare three methods of costing end-of-life care for a sample of men in the Cluster randomised triAl of PSA testing for Prostate cancer (CAP)). The first method was based on resource-use data collected through medical record review, with costs applied via national reference costs. The second method utilised routinely collected HES data, with national reference costs applied. A third comparative set of information on cost data was sought from finance departments. The analysis was restricted to the inpatient component of the total costs, as this is known to be a key healthcare cost driver.^18^

## Methods

### Selection of patients and events

The CAP Trial (ISRCTN92187251) is evaluating the effectiveness and cost-effectiveness of prostate-specific antigen (PSA) testing with a primary outcome of prostate cancer mortality. General practices in eight centres within the UK (Bristol, Cardiff, Cambridge, Leeds, Newcastle, Birmingham, Sheffield and Leicester) were allocated to either population-based PSA testing as part of the ProtecT trial or usual practice (the provision of an informed choice to men seeking advice about the test).^19^ Participants were all men aged 50–69 years who were registered with one of the study practices. As part of the CAP Trial, a detailed review of medical records was conducted for men who were diagnosed with prostate cancer or died with prostate cancer or bone metastases according to the death certificate.^19^

Inpatient resource-use data for a convenience subsample of men from the CAP Trial were made available in order to carry out this methodological study. The sample included men in CAP who (1) had a diagnosis of prostate cancer notified by the Health and Social Care Information Centre (HSCIC) and confirmed in medical records, (2) died of prostate cancer or other causes at least a year after first being investigated for prostate cancer and (3) had undergone the complete medical record review process. As such, they represent a homogeneous, but arbitrary rather than truly random, sample that excludes men who died rapidly following diagnosis. Men from each of the seven CAP centres in England (Bristol, Birmingham, Cambridge, Newcastle, Sheffield, Leeds and Leicester) were included and were drawn from intervention and usual care arms of the trial, with the researcher (JCT) blinded to trial arm. Descriptive statistics including age, index of multiple deprivation (IMD, an area-level measure of deprivation^20^) and cause of death were obtained.

### Measurement of resource use

Inpatient resource use was identified using two different sources. All inpatient events whether related to prostate cancer or not were identified by a detailed review of medical records conducted by trained research assistants. Start and end dates for each event were recorded along with the type of ward in which the event took place. HES inpatient records, including episode start dates, durations and HRG V.3.5 codes,^21^ were also available. Day cases were excluded from the analysis because there is ambiguity over their definition.^22^ In the absence of HES data describing the type of stay, HES day cases were defined as stays of zero nights. Hospice care was also excluded from the analysis because it is often provided outside the NHS and is not recorded in HES.

### Application of costs

For resource use identified in medical record review, costs were assigned on the basis of the type of ward in which the inpatient event had occurred and the length of stay. Costs were applied using the publicly available NHS reference costs, which include both direct and indirect costs.^23^ Ward types for each event were mapped to service codes describing the specialty under which the man was treated in the NHS reference costs. For example, urology has a service code of 101, while cardiology is represented by 320. For each of the listed service codes, median costs for a patient's overnight stay were derived from a data set containing organisation-specific costs for 2010/2011.^24^ Elective and non-elective inpatient (both short and long) stays were included in the averages because information on whether the stay was elective or not was not available for inpatient events in the medical record review data set; primary care trust data and outsourced events were excluded as we were interested in NHS secondary care costs. Where the ward type was unknown or unclear in the medical records, mapping to service codes was not possible. Mean imputation was used in these cases: a mean cost per night (weighted by the number of nights) was derived from all episodes with available cost data, and applied to events with missing costs. Sensitivity to the imputed value was explored by using the maximum and minimum costs.

For resource use identified via HES records, HRG V.3.5 codes were used to apply costs directly from the 2005/2006 NHS reference costs (the most recent year for which HRG V.3.5 costs were published) on the basis of individual episodes. Elective and non-elective admissions were taken into account. A *per diem* costing was applied to excess bed days beyond the standard number of days anticipated for a given HRG (known as the ‘trim point’^25^). Costs were inflated to 2010/2011 levels using the hospital and community health services index.^26^

A third comparison was made by requesting cost data from trust finance departments for a small number of men whose medical record review was completed earliest. Trusts in each of the eight areas participating in CAP were sent a tailored questionnaire designed to minimise the burden on respondents by only requesting information relating to events that occurred within that trust. For example, trusts were only asked to supply the cost of a patient's overnight stay in a cardiology ward if a man in that trust had used the resource; this approach resulted in questionnaires of varying length. Questionnaires were emailed to named contacts identified by CAP researchers from information about the finance departments on each trust's website, with a request to return the information within 4 weeks. Periodic reminders were sent by email over a period of 5 months, and departments were contacted by telephone in order to ascertain whether there might be any problems with fulfilling the request.

### Analysis

Statistical analysis was carried out using Stata V.12 (StataCorp. Stata Statistical Software: Release 12. College Station, TX: StataCorp LP, 2011). For medical record review resource use, NHS reference costs were assigned to the month prior to death in which they occurred on a *per diem* basis; for long stays, therefore, costs could fall in more than 1 month. For HES-identified resources, costs were assigned to the month in which the first day of the episode occurred to reflect the fixed-cost nature of HRGs. Inpatient cost profiles were derived for mean costs incurred on a monthly basis over the last year of life.

Total mean resource-use costs over the final year of life were also calculated for the two methods of collecting resource-use data costed using NHS reference costs, and differences were derived for each month prior to death. Two-sample paired t tests were used to derive p values for the differences between costs; a 1% significance level is appropriate for the multiple tests carried out on these data. A manual inspection of inpatient events occurring in medical record review and HES records was conducted, with the aim of identifying missing events in either data set.

## Results

### Data sets

HES and medical record review data were available for 292 men, representing seven centres participating in CAP in England. The men died between 2004 and 2012 at ages 52–78 years (mean (SD) age at death: 68.2 (5.4)). They had been diagnosed with prostate cancer at a mean (SD) age of 65.6 (5.3) years, and lived for a mean of 2 years and 8 months from diagnosis until death (SD=18 months). Two hundred and sixty-two of the men had undergone assessment by a committee set up to determine cause of death from data collected during medical record review;^19^ of these, 161 men (61.5%) were assessed as having died of prostate cancer. Of the remaining men, 64 (24.4%) had another cancer, 13 (5.0%) had cardiovascular disease and 24 (9.2%) had other causes cited on the death certificate. The men had a mean (SD) IMD score of 25.0 (17.3). Costs were imputed for 362/7722 (4.7%) of nights in the medical record review data set.

Comparative costs were requested from trust finance departments for a subset of 88 of these men, via questionnaires varying in length between three and seven pages. Full sets of requested costs were received from three out of the seven finance departments that were contacted, with response times ranging from 9 to 106 days; the shortest questionnaire resulted in the fastest response. A further partial set was received from a fourth department after a period of 125 days. Three departments had been unable or unwilling to supply costs after a period of 6 months. One department returned an extract from the NHS reference costs, and the remaining three varied in terms of the factors included in the costs. Owing to the poor response rate and inconsistencies in reporting, the strategy was not considered successful and no further data were requested.

### End-of-life inpatient cost profiles

**Figure 1:**
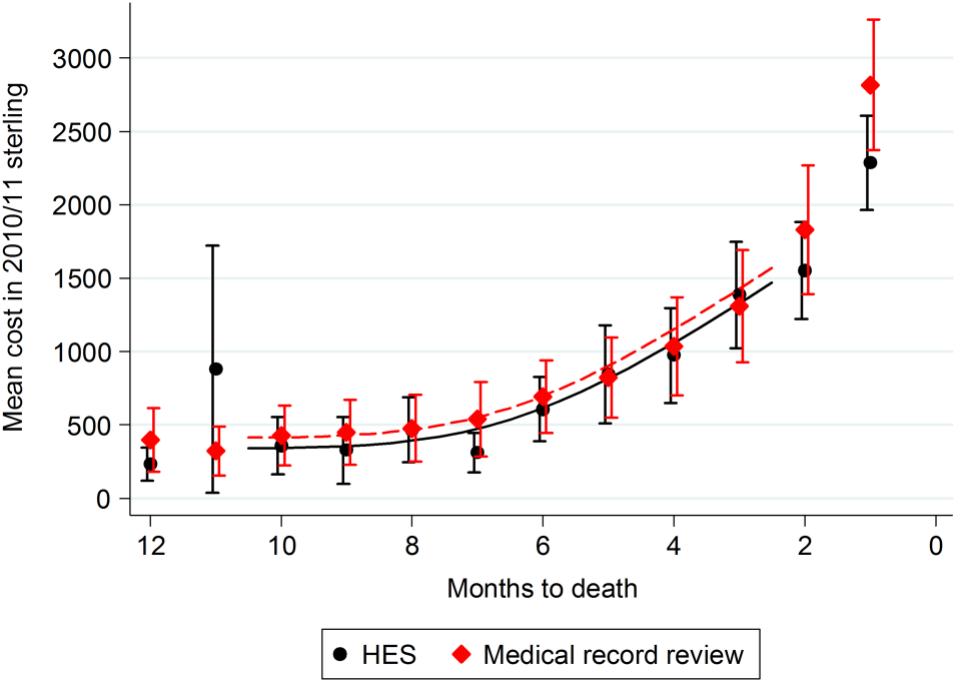
End-of-life inpatient cost profiles for men who have been diagnosed with prostate cancer; resource use was identified through (A) medical record review and (B) Hospital Episode Statistics (HES). National reference costs were used to assign costs to the resource use.

Month-by-month inpatient cost profiles over the final year of life were derived using resource-use data from HES and medical record review costed with NHS reference costs (figure 1). The profiles were similar to one another and follow the expected pattern of a substantial rise in costs in the final months leading up to death. The 95% CIs overlapped at each month, and there was no discernible pattern that might suggest the existence of a systematic error between the two costing methods. The inpatient cost profiles diverged slightly as death approached, but the values were not significantly different at the 1% significance level. The wider variation in month 11 prior to death arose from a single man with a very lengthy stay beyond the trim point in the HES data set. Insufficient data returned from finance departments precluded the derivation of an inpatient cost profile using this costing methodology.

### Mean resource use per patient

The mean resource use per man over the final year of life was £11 122 (95% CI £9083 to £13 161) using medical record review and national reference costs, and £10 223 (95% CI £8880 to £11 565) using HES data with reference costs. Costs associated with HES data were slightly lower (about 8%) than those associated with medical record review data, but the difference was compatible with chance (p=0.3). Sensitivity analysis indicated that this result was not sensitive to the imputed value used in the medical record review costing; all CIs remained overlapping.

### Event identification

The definition of a single event was not consistent between the two sets of resource use, with some episodes recorded as single events in medical record review data appearing as multiple events in HES and vice versa. Therefore, events in the data sets were not congruent; minor differences in dates and lengths of stay were also common. However, there was no ambiguity where men had zero events recorded in one or the other data set. Eleven men (3.8%) for whom events were recorded in HES had all these events missing from medical record review; similarly, 7 men (2.4%) with no events according to HES had events identified in medical record review.

## Discussion

The end-of-life inpatient cost profiles derived using HES and medical record review data were broadly similar. Using HES data resulted in slightly lower costs overall than using medical record review data. HES costs represented ∼92% of medical record review costs, although there was no evidence of a true difference. This suggests that it should be possible to use either of the two costing methods in randomised controlled trials. It is unlikely that the discrepancy in costs can be explained by events not having been captured in HES. Eleven men had events in HES but no events picked up by medical record review; this is likely to have arisen as a result of difficulties tracing events that may have occurred in hospitals not approached during the medical record review process. However, seven men had events identified in medical record review and no events in HES, suggesting that HES does not represent a completely accurate gold standard.

Requesting data from finance departments proved very difficult, and was ultimately an unsuccessful approach. This may have been linked to the volume of information being requested, as the fastest turnaround arose with the shortest questionnaire. In the absence of a formal responsibility for trusts to supply the data, responding to a request appeared to be low on a list of priorities. The trusts all appeared to have different ways of working, and for some it appeared that the questions asked were not meaningful. Although trusts were asked to identify the different types of costs included in their estimates, it was not always apparent that the data supplied were directly comparable in terms of, for example, inclusion of procedures undergone during a hospital stay. The low response rate would have resulted in a large degree of estimation for missing data, and makes the finance department method unsuitable for a CEA.

The HRG system is used to drive reimbursement and create incentives for providers; as such, it does not necessarily reflect the true opportunity costs of the resources consumed. The nature of averages means that hospitals will be overcompensated for some procedures and underpaid for other, more costly, procedures. It is possible that the distribution of underpaid costly procedures is concentrated in the final year of life which would accentuate any underpayment recorded. Inevitably, however, a *per diem* costing method with a cost applied for each day in hospital places considerable emphasis on the length of stay, with the same costs attributed to the first and all subsequent days.^27^ This may not reflect reality well, as it is more likely that there are variable living and medical costs throughout an episode with higher costs at the start of an episode.

A study based on Scottish data has looked at five different methods of costing hospital inpatient stays, comparing two DRG-based costing methods and three methods incorporating a *per diem* approach.^27^ The authors found substantial differences in overall costs depending on the costing method employed with higher costs observed when using a *per diem* approach, and recommended using an HRG-based costing method. However, the study was restricted to acute episodes, and did not use the English HES data set. Microcosting approaches attempt to identify and include all relevant costs in some detail; in preliminary work, Dakin *et al*^28^ found that using HRG data resulted in lower costs than microcosting in the context of a trial studying age-related macular degeneration. A comparison of a DRG-based costing system with microcosting in Ireland concluded that for disease areas with high-cost treatments, DRG estimates do not yield reliable results and microcosting should be considered.^29^ Our results add to this body of literature and suggest that HRG-based costing methods are appropriate in some instances.

The opportunity to reduce the cost of research through the use of HES appears to be a compelling argument in favour of adopting this approach for studies that require data extraction on large numbers over a wide geographical area. Medical record review is currently estimated by the CAP team to take 4–6 h per review, and can take as long as 28 h. It would be unrealistic to undertake this very time-consuming process for a large number of participants. Medical record review also has the potential to overlook events that occur at hospitals outside those visited by researchers. However, there are some limitations associated with using HES data for trial purposes. Coverage is restricted to England; therefore, studies with participants in other parts of the UK will need to employ further methods to supplement the HES data. Also, HRGs do not have the cost discrimination required to differentiate between, for example, two similar surgical procedures; in this instance, a microcosting approach based on asking finance departments for costs would still be necessary. For trials with long-term follow-up, there are also specific problems relating to changes in administrative methods over the years; for example, high-cost items were included in base HRGs in V.3.5, but were unbundled and assigned their own codes in HRG V.4.0.^30^

The study has strengths, but also some limitations. The strengths included the fact that we analysed the data retrospectively, so it was possible to define the final year of life accurately. Research assistants received twice yearly training and review of their performance, so we are confident that the data collection process in medical record review was meticulous and comprehensive. However, conclusions that can be reliably drawn from this study are constrained by some limitations. Although there is no reason to believe that any biases exist, the sample was not selected completely at random. There is a lack of clarity over the definition of a day case, with some trusts classifying the same activity as an outpatient event. This may have led to day cases being inaccurately identified either in HES or medical record review^22^; day case events were excluded from the study, but the confusion may have led to some attribution errors. The men included in this study were slightly more deprived on average (IMD score=25.0) than the general population of men in CAP (IMD score=22.9). Men who died rapidly following diagnosis (within a year) were excluded; therefore, the study population is not a general one. Mean imputation was used for missing costs in the medical record review data, reducing the variance; however, this does not affect the conclusions of the study. The findings in this study relate to men diagnosed with prostate cancer, and it is uncertain whether these findings are generalisable to other cancers or conditions. We do not know whether the inpatient costs in the final year of life vary according to whether the man died of prostate cancer or of another cause. For men dying of prostate cancer, the cost of inpatient care in the final year of life has been estimated at ∼£7500,^31^ slightly lower than our estimate for all men; we intend to explore this issue using our data in the future when the sample size is larger to allow precise estimation of stratified costs.

The purpose of this study was to investigate different costing methods that could be employed in clinical trials, and to validate the use of the HES inpatient data set in particular; the costs derived should not, therefore, be interpreted as end-of-life costs. The study was restricted to inpatient resource use;^18^ however, informal caregiving,^32^ chemotherapy^33^ and hospice care^34^ may also be substantial in the end-of-life period. Derivation of an overall cost of end-of-life prostate cancer care is planned for future work.

## Conclusions

Using HRGs from HES data coupled with NHS reference costs did not result in significantly different derived costs when compared with medical record review costing methods. This suggests that it should be acceptable to use routinely collected HES data in economic evaluations conducted alongside randomised controlled trials, although replication in alternative contexts would be valuable. Neither HES nor medical record review represent gold standards of resource-use data collection, with some inpatient events missed from both data sets.

If data from medical record review are to be used, applying national reference costs is the preferred costing method as it is considerably less time consuming than contacting finance departments, and results in a substantially more complete data set. Researchers intending to use data from finance departments should make contact well in advance of requiring the data and anticipate a low response rate.
